# Neither pre-sleep nor post-exercise protein consumption influences resistance exercise training adaptations in older adults

**DOI:** 10.1080/15502783.2025.2519511

**Published:** 2025-06-20

**Authors:** Alex O. Klemp, Michael J. Ormsbee, Mingchia Yeh, Chester M. Sokolowski, Do-Houn Kim, Lynn B. Panton, Jeong-Su Kim

**Affiliations:** a Florida State University, Department of Nutrition and Integrative Physiology, Tallahassee, FL, USA; b Florida State University, Institute of Successful Longevity, Tallahassee, FL, USA; c Florida State University, Institute of Sports Sciences and Medicine, Tallahassee, FL, USA; d University of KwaZulu-Natal, School of Health Sciences, Discipline of Biokinetics, Exercise and Leisure Sciences, Durban, South Africa; e Slippery Rock University, Department of Exercise Science, Slippery Rock, PA, USA; f Gonzaga University, Department of Kinesiology and Sport Management, Spokane, WA, USA

**Keywords:** Resistance exercise, protein, older adults, hypertrophy, muscle strength, muscle thickness

## Abstract

**Purpose:**

Limited data exists that compare pre-sleep versus post-exercise protein intake during resistance exercise training (RET) in older adults. This study examined whether 40 g of protein consumed post-exercise (PRP) or pre-sleep (PSP) enhances muscle thickness (MT) and strength compared to RET alone (RETO) in older men.

**Methods:**

Thirty untrained older men (65.7 ± 4.0 yrs) completed 12 weeks of supervised RET (2×/week) and were randomized to PRP (*n* = 9), PSP (*n* = 11), or RETO (*n* = 10). MT of the vastus lateralis (VL), rectus femoris (RF), and vastus intermedius (VI) and 1-repetition maximum (1-RM) for leg and chest press were assessed at weeks 0, 6, and 12.

**Results:**

VL (0 to 12 weeks: + 0.16 cm, 95% C.I. [0.06, 0.25]), RF (0 to 12 weeks: + 0.13 cm, 95% C.I. [0.03, 0.23]), and VI MT (0 to 12 weeks: + 0.18 cm, 95% C.I. [0.05, 0.31]) and chest press (0 to 12 weeks: + 10.9 kg, 95% C.I. [5.50, 16.3]) and leg press (0 to 12 weeks: + 28.3 kg, 95% C.I. [19.63, 37.1]) 1-RM increased (*p* < 0.050) with no group differences.

**Conclusion:**

Consuming 40 g of protein post-exercise or pre-sleep did not enhance RET-induced improvements in muscle thickness or strength in older adults with adequate baseline protein intake (≥1.0 g/kg/day). RET alone elicited significant gains, emphasizing that adherence to training and meeting daily protein requirements are more critical than timing strategies for untrained older adults. ClinicalTrials.gov identifier: NCT05922475, 06/23/2023, retrospectively registered.

## 1. Introduction

Aging is associated with progressive declines in lean mass, muscular strength, and power, but resistance exercise training (RET) combined with adequate dietary protein intake can mitigate these losses [1]. While the importance of total daily protein intake is well-established, the timing of protein consumption, particularly its proximity to exercise, remains equivocal. Traditional guidelines emphasize post-exercise protein ingestion to maximize muscle protein synthesis (MPS) and RET adaptations; however, empirical support is lacking [2]. As such, pre-sleep protein intake – consuming protein 30 minutes before nocturnal sleep – may offer unique advantages, as overnight muscle protein synthesis (MPS) appears to be lower than morning fasted rates [3]. Indeed, ingestion of protein prior to nocturnal sleep has been shown to increase MPS levels [4] without adversely affecting metabolic health markers (e.g. blood lipids, glucose, or insulin; [5]). Furthermore, consumption of a greater amount of protein in the evening, compared to earlier in the day, has been shown to improve lean mass accretion, even when total daily protein is matched [6]. These findings position pre-sleep protein as a promising strategy to amplify RET-induced adaptations, particularly in older adults who face age-related anabolic resistance [3].

Nonetheless, the acute effects of pre-sleep protein on post-exercise recovery are mixed. For example, consuming 25 g of protein after evening resistance exercise improved recovery of isometric knee extension strength and muscular endurance in young adults [7], whereas 40 g of protein failed to enhance 1-repetition maximum (1-RM) strength recovery 12 hours post-exercise in a similar cohort [8]. In longer-term interventions, pre-sleep protein supplementation during 12 weeks of RET increased strength and muscle hypertrophy in young adults compared to a placebo [9]. Although data on pre-sleep protein consumption in older adults during RET are limited [10], the benefits do not appear to be similar to those observed in younger adults as neither pre-sleep, nor post-exercise, protein consumption augmented RET adaptations in older adults [11]. However, this was possibly due to insufficient dosing, as that study provided only 21 g of protein, falling short of the recommended 40 g to optimally stimulate overnight MPS and overcome anabolic resistance in older populations [3,12]. Thus, the efficacy of pre-sleep protein in older adults remains uncertain, warranting further investigation with optimal protein dosages.

The primary aim of this study was to compare the effects of pre-sleep protein intake versus traditional post-exercise protein intake on muscle hypertrophy and strength during a 12-week RET program in older adults. A third RET-only group (no additional supplemental protein) served as a control. We hypothesized that both protein timing strategies (post-exercise and pre-sleep) would elicit greater improvements in muscle strength and hypertrophy than RET alone, with no significant differences between the two protein conditions.

## 2. Methods

### 2.1. Study design

This study was a 12-week, double-blind, randomized, placebo-controlled trial. Participants were randomized (www.randomizer.org) into three groups using a covariate-adaptive method to ensure balance in baseline protein intake: (1) a post-exercise protein group (PRP, *n* = 9), which consumed 40 g of protein immediately after RET sessions; (2) a pre-sleep protein group (PSP, *n* = 11), which consumed 40 g of protein 30 minutes before bedtime; and (3) a RET-only control group (RETO, *n* = 10), which received no supplemental protein. All groups performed identical, supervised full-body resistance training programs twice weekly by research personnel and certified trainers. Quadriceps muscle thickness and 1-repetition maximum (1-RM) strength for machine leg press and chest press were measured at baseline (Week 0), mid-intervention (Week 6), and post-intervention (Week 12). The data presented in this study are a subset of a larger project with additional outcome measures. The protocol for the additional outcome measures can be found at ClinicalTrials.gov (identifier: NCT05922475) and data are available at the Open Science Framework repository (https://osf.io/ae5un/?view_only=4dcee5e2845a4304936dc5f05c2b158c).

Baseline testing occurred over 2–3 visits. During the first visit, participants arrived in the morning after a 12-hour fast to complete informed consent, health history questionnaires, cognitive screening (Montreal Cognitive Assessment), anthropometric measurements (height, weight), and muscle thickness scans. Participants were also provided with a 3-day dietary record and received counseling to improve accuracy. A second visit 72–96 hours later included 1-RM familiarization for the leg press and chest press machines, followed by dietary record submission and review. A third visit 5–7 days later consisted of baseline chest press and leg press 1-RM testing. Some participants opted to complete the initial 1-RM testing at Visit 2, reducing their baseline testing to two visits. Follow-up testing at Weeks 6 and 12 mirrored baseline procedures, and dietary records were collected at each testing session. Participants were instructed to avoid vigorous activity for 48 hours before testing.

### 2.2. Participants

Thirty-two sedentary (not exercising regularly for the past 6 months) males aged 60–75 years were enrolled in this study. Participant characteristics are summarized in Table 1. Exclusion criteria included: (1) cardiovascular, metabolic, or renal disease; (2) uncontrolled hypertension (resting blood pressure ≥ 140/90 mmHg); (3) orthopedic limitations affecting exercise participation; (4) skeletal muscle disorders or milk protein allergy; (5) psychiatric conditions or psychotropic medication use; (6) cognitive impairment (Montreal Cognitive Assessment [MoCA] score < 26); (7) active attempts to gain/lose body mass; or (8) body mass index (BMI) ≥35 kg/m^2^. Eligibility was determined via health history questionnaires, physical activity assessments, MoCA screening, and anthropometric measurements (height, weight). All participants provided written informed consent approved by the Florida State University Institutional Review Board (#2018.25996). This study was registered with ClinicalTrials.gov (identifier: NCT05922475, 06/23/2023, retrospectively registered).

**Table 1.** Subject characteristics.

	RETO (n = 10)	PRP (n = 9)	PSP (n = 11)
Age (yrs)	65 ± 5	67 ± 4	66 ± 4
Height (cm)	178.3 ± 9.1	175.3 ± 7.4	176.7 ± 10.6
Body mass (kg)	85.4 ± 13.4	82.9 ± 12.8	88.8 ± 13.7
Leg press 1RM (kg)	157 ± 17	152 ± 36	158 ± 36
Chest press 1RM (kg)	159 ± 30	152 ± 36	165 ± 47

RETO = Resistance exercise training only and no additional protein; PRP = post-resistance exercise training protein; PSP = pre-sleep protein; yrs = years; cm = centimeters; kg = kilograms; 1RM = one-repetition maximum. Data presented as mean ± standard deviation.

### 2.3. Baseline testing

#### 2.3.1. 1^st^ baseline visit

##### 2.3.1.1. Body mass and height

Body mass (BM, kg) and height (cm) were measured by a digital scale and stadiometer (SECA, Hamburg, Germany), respectively.

#### 2.3.2. Muscle thickness

Muscle thickness (MT) was assessed at Weeks 0, 6, and 12 using B-mode ultrasonography (HD11 XE, Philips, Netherlands) with a 3–12 MHz linear-array transducer (L 12–3, Philips, Netherlands) to evaluate quadriceps hypertrophy. Participants rested supine for 10 minutes prior to scanning to minimize fluid shifts. Anatomical landmarks were marked with a water-soluble pen at the midpoint between the greater trochanter and lateral femoral condyle (vastus lateralis and intermedius) and at the midpoint between the anterior superior iliac spine and the superior patellar border (rectus femoris). These anatomical locations allowed for imaging of the vastus lateralis, vastus intermedius, and rectus femoris muscles.

All scans were performed by the same researcher who applied minimal transducer pressure to avoid tissue compression. Muscle thickness was defined as the distance (cm) from the adipose-muscle interface to the muscle-bone interface. The measurements from the vastus lateralis, vastus intermedius, and rectus femoris were summed for a total quadriceps thickness measurement. The intraclass correlation coefficient (ICC: mean-rating of two measurement, two-way mixed effect, absolute agreement), standard error of measurement (SEm), and coefficient of variation (CV) with 95% confidence intervals (CI) were calculated for the vastus lateralis (ICC = 0.99, 95% CI [0.94, 0.99]; SEm = 0.01 cm, 95% CI [0.06, 0.26]; CV = 3.31% [1.98, 9.53]), rectus femoris (ICC = 0.99, 95% CI [0.99, 0.99]; SEm = 0.05 cm, 95% CI [0.03, 0.15]; CV = 1.87% [1.10, 5.3]), and for the vastus intermedius (ICC = 0.99, 95% CI [0.92, 0.99]; SEm = 0.12 cm, 95% CI [0.08, 0.38]; CV = 5.97% [3.57, 17.30]).

#### 2.3.3. Food record and analysis

Dietary intake was assessed at baseline, Week 6, and Week 12 using 3-day food records (two weekdays and one weekend day) for analysis of macronutrient and energy intake. Participants were instructed to maintain their usual dietary habits throughout the study, with the exception of consuming their assigned protein or placebo supplement. To enhance accuracy, they were provided with written instructions and encouraged to photograph meals and snacks. Records were reviewed in person with a researcher upon submission to resolve any issues. Food records were analyzed using the MyFitnessPal® phone application. Macronutrient data (protein, carbohydrate, fat) and total energy intake (kcal/day) were averaged across the three recording days at each timepoint.

#### 2.3.4. 2^nd^ & 3^rd^ baseline visit

##### 2.3.4.1. One-repetition maximum (RM) strength

Participants underwent 1-RM testing at weeks 0, 6, and 12 for the leg press and chest press exercises. Before each 1-RM test, participants completed five minutes of light cycling on a stationary cycle (Monark 828E, Langley, WA) followed by a full body dynamic warm-up that consisted of various body weight movements lasting approximately five minutes. Thereafter, leg press 1-RM procedures began with a specific warm-up consisting of a loaded leg press for 10 repetitions at 30%, five repetitions at 50%, two repetitions at 75%, and one repetition at 85% of estimated 1-RM. All warm-up sets were completed with 1-minute rest periods. Subsequent 1-RM attempts began at 95% of the estimated 1-RM and increased incrementally with 3-minute rest periods between each attempt. 1-RM testing continued until the participants could no longer complete a full repetition with the correct form. After leg press 1-RM was established, the same procedures were followed for the chest press exercise.

### 2.4. Resistance exercise training (RET) protocol

All participants completed 12 weeks of RET, two times per week on nonconsecutive days (separated by 72–96 hours), which has been demonstrated to be efficacious for increasing maximum muscle strength and hypertrophy in older adults [13–15]. Participants generally completed their RET sessions at consistent days and times throughout the study between 0700 and 1600. Research personnel and certified personal trainers supervised all sessions. Each session began with a 5-minute cycling warm-up (Monark 828E, Langley, WA) at 50 rpm with 0.5 kg resistance, followed by a dynamic bodyweight warm-up.

The RET program included three lower-body exercises (leg press, leg extension, leg curl) and five upper-body exercises (chest press, shoulder press, latissimus row, triceps dip, biceps curl), performed for 3–4 sets of 4–12 repetitions at 50–80% of 1-RM (Table 2). Primary exercises (leg press, chest press) followed 1-RM-based loading, which was adjusted after Week 6 1-RM testing. Accessory exercises utilized a repetition-in-reserve (RIR) scale, with the goal to reach an RIR of 2–3 after each set [16]. Rest intervals were three minutes between each set. If participants were not able to complete the prescribed number of repetitions within a set, the training load for the subsequent sets of that specific exercise was reduced by ~ 2.5% for each missed repetition.

**Table 2.** Overview of exercise training program.

Main Exercise: Chest and Leg Press		
Week	Day 1	Day 2
1	2x12 @ 50%	2x10 @ 55%
2–3	3x12 @ 60%	3x10 @ 65%
4	3x11 @ 62.5%	3x9@67.5%
5–6	3x10 @ 65%	3x8 @ 70%
7	2x2 @ 80%, 1 × 1@ 85%	Mid 1-RM test
8–9	3x8 @ 70%	3x6 @ 75%
10–11	3x6 @ 75%	4x4 @ 80%
12	2x2 @ 80%, 1 × 1@ 85%	Post 1-RM test

RM = Repetition Maximum.

### 2.5. Protein supplementation

Participants in the PRP and PSP groups consumed a 40 g protein supplement (Dymatize® Elite XT), comprising a 4:1 whey-to-casein blend (265 kcal: 40 g protein, 15 g carbohydrates, 5 g fat. Protein sources included whey protein concentrate, milk protein isolate, whey protein isolate, micellar casein). This is the recommended amount of protein for older adults and the amount to stimulate overnight MPS [3,12]. The placebo was a non-caloric powder matched for flavor and texture and was prepared by investigators uninvolved in outcome assessments, along with the protein supplement. Both supplements were placed into individual storage bags within identical, unlabeled containers to ensure blinding, and participants received daily compliance sheets to log consumption.

The PRP group consumed the protein supplement immediately post-exercise and the placebo 30 minutes before sleep, while the PSP group ingested the placebo post-exercise and the protein supplement pre-sleep. The RETO group served as a control and received no supplementation, and therefore, was not blinded to their group assignment. All participants fasted for at least two hours before and after exercise sessions, except for consuming their assigned post-RET supplement alongside a 190-kcal snack bar (Nature Valley™ Oats ’N Honey). Pre-sleep supplements were taken at least 2 hours after the last meal, and on non-exercise days, supplements were consumed between breakfast and lunch to ensure consistent daily intake. Compliance was verified through returned supplement containers and review of compliance logs.

### 2.6. Statistical analysis

Baseline differences between groups for participant characteristics, strength measures, dietary intake, and training volume were assessed using a linear regression. Changes in body mass, muscle thickness, and 1-RM during RET were analyzed with linear-mixed effect models optimized with restricted maximum likelihood. The fixed factors were time (Week 0, Week 6, Week 12) and group (PRP, PSP, RETO), with random intercepts for participants to account for repeated measures. Degrees of freedom and p-values were estimated with the Kenward-Roger method. Significance was set at *p* < 0.05. For significant effects, pairwise comparisons were performed with Sidak adjusted p-values and CIs. Results are reported as estimated marginal means (EMMs) with 95% CIs for pairwise comparisons and arithmetic mean ± standard deviation (SD) for all other data. Normality was assessed via quantile-quantile plots, and violations are noted where applicable.

A sensitivity power analysis was conducted to determine the minimum detectable effect size (f) for the group × time interaction, using the following parameters: α = 0.05, power = 0.80, sample size = 30, 3 groups, 3 measurements, and nonsphericity correction coefficient = 1. The analysis resulted in an effect size of 0.66, corresponding to raw changes of ~0.21 cm for total muscle thickness, and ~11 kg in total 1-RM, calculated using the residual standard deviation from the respective models. All analyses were performed with R Statistical Software (R Core Team, version 4.4.0).

## 3. Results

### 3.1. Participants

**Figure 1.**
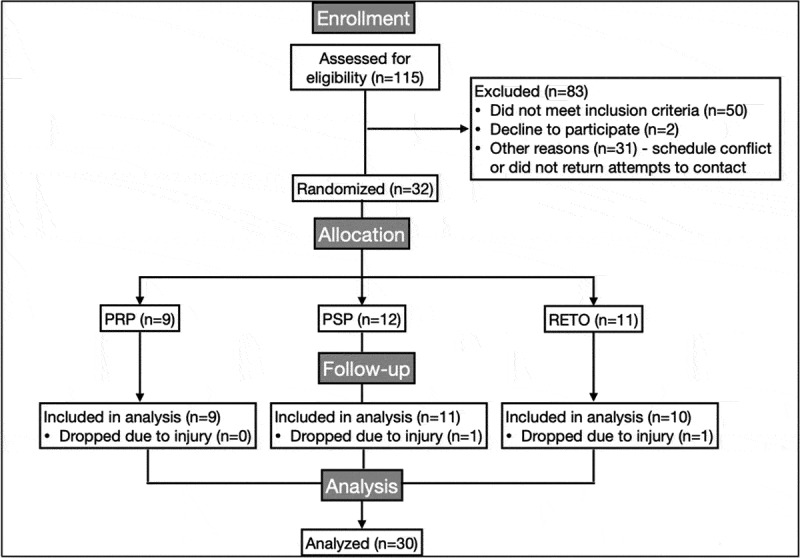
CONSORT flow chart. PRP = post-resistance exercise training protein. PSP = pre-sleep protein. RETO = resistance exercise training only and no additional protein.

Thirty participants completed the study (PRP: *n* = 9, PSP: *n* = 11, RETO: *n* = 10; Figure 1). At baseline, there were no significant differences in age, body mass, leg press 1-RM, or chest press 1-RM. Total training volume over the 12-week intervention did not differ significantly among groups (PRP: 602,253 ± 114,656 kg; PSP: 655,906 ± 170,419 kg; RETO: 627,322 ± 67,374 kg). Compliance was high, with participants attending 96% of supervised exercise sessions and consuming 97% of assigned supplements.

### 3.2. Macronutrient intake

At baseline, there were no significant differences between groups in macronutrient intake (carbohydrates, fat, protein), total energy intake (kcal/day), or protein intake normalized to body mass (g/kg/day; see Table 3).

**Table 3.** Macronutrient intake.

	RETO (n = 10)			PRP (n = 9)					PSP (n = 11)				
	Week 0	Week 6	Week 12	Week 0	Week 6	Week 6 + sup	Week 12	Week 12 + sup	Week 0	Week 6	Week 6 + sup	Week 12	Week 12 + sup
Absolute consumption													
CHO (g∙d^−1^)	239 ± 87	226 ± 101	234 ± 83	231 ± 44	211 ± 59	226 ± 59	229 ± 82	244 ± 82	241 ± 33	248 ± 59	263 ± 59	242 ± 41	257 ± 41
Fat (g∙d^−1^)	89 ± 22	89 ± 40	109 ± 49	90 ± 25	88 ± 18	93 ± 18	89 ± 29	94 ± 29	111 ± 35	103 ± 34	108 ± 34	92 ± 28	97 ± 28
PRO (g∙d^−1^)	91 ± 24	91 ± 28	94 ± 30	85 ± 22	80 ± 20	120 ± 20	87 ± 20	127 ± 20	97 ± 13	92 ± 24	132 ± 24	82 ± 16	122 ± 16
Energy (kcal∙d^−1^)	2,182 ± 294	2,125 ± 454	2,389 ± 546	2,125 ± 342	1,962 ± 388	2,227 ± 388	2,086 ± 542	2,351 ± 542	2,397 ± 340	2,336 ± 394	2,601 ± 394	2,158 ± 410	2,423 ± 410
Relative consumption													
CHO (g∙kg^−1^∙d^−1^)	2.9 ± 1.1	2.7 ± 1.4	2.7 ± 0.9	2.8 ± 0.7	2.5 ± 0.4	2.7 ± 0.4	2.7 ± 0.8	2.9 ± 0.7	2.8 ± 0.6	2.8 ± 0.8	3.0 ± 0.9	2.8 ± 0.7	3.0 ± 0.7
Fat (g∙kg^−1^∙d^−1^)	1.0 ± 0.2	1.0 ± 0.3	1.3 ± 0.5	1.1 ± 0.3	1.1 ± 0.2	1.1 ± 0.2	1.1 ± 0.4	1.2 ± 0.4	1.3 ± 0.4	1.1 ± 0.3	1.2 ± 0.3	1.1 ± 0.3	1.1 ± 0.3
PRO (g∙kg^−1^∙d^−1^)	1.1 ± 0.3	1.0 ± 0.3	1.1 ± 0.3	1.0 ± 0.3	1.0 ± 0.2	1.5 ± 0.3	1.1 ± 0.3	1.6 ± 0.3	1.1 ± 0.3	1.0 ± 0.3	1.5 ± 0.3	0.9 ± 0.2	1.4 ± 0.3
Energy (kcal∙kg^−1^∙d^−1^)	25.6 ± 4.0	25.0 ± 6.6	27.6 ± 5.6	26.1 ± 5.8	23.7 ± 3.6	27.0 ± 3.7	25.5 ± 7.2	28.8 ± 7.4	27.6 ± 5.7	26.2 ± 5.6	29.1 ± 5.9	24.8 ± 6.0	27.8 ± 6.2

RETO = Resistance exercise training only and no additional protein; PRP = post-resistance exercise training protein; PSP = pre-sleep protein; SUP = protein supplement; CHO = carbohydrates; g = gram; d = day; PRO = protein; kcal = kilocalories; kg = kilograms. Data presented as mean ± standard deviation.

Analyses of macronutrient intake (excluding supplemental protein) revealed a significant group × time interaction for fat intake relative to body mass (F(4,50.3) = 2.589, *p* = 0.048), though post hoc pairwise comparisons showed no significant changes over time. No other interactions or main effects were observed for carbohydrates, total fat, or energy intake.

When including supplemental protein, significant group ×time interactions emerged for absolute protein intake (F(4,50.4) = 5.112, *p* = 0.002) and protein intake per kg body mass (F(2,50.3) = 17.668, *p* < 0.001). Both PRP and PSP groups exhibited significant increases in absolute protein intake from Week 0 to Week 6 (PRP: Δ = 35.48 g/day, 95% CI [15.87, 55.09], *p* < 0.001; PSP EMM = 35.17 g/day, 95% CI [17.57, 52.77], *p* < 0.001) and Week 0 to Week 12 (PRP: EMM = 41.65 g/day, 95% CI [22.80, 60.49], *p* < 0.001; PSP: EMM = 24.92 g/day, 95% CI [7.88, 41.97], *p* = 0.010), whereas RETO showed no changes. Similar patterns were observed for protein intake per kilogram of body mass, with PRP and PSP increasing from Week 0 to Week 6 (PRP: EMM = 0.42 g/kg/day, 95% CI [0.19, 0.66], *p* < 0.001; PSP: EMM = 0.37 g/kg/day, 95% CI [0.16, 0.58], *p* < 0.001) and Week 0 to Week 12 (PRP: EMM = 0.51 g/kg/day, 95% CI [0.28, 0.73], *p* < 0.001; PSP: EMM = 0.28 g/kg/day, 95% CI [0.08, 0.48], *p* = 0.004). Mean values and SDs are presented in Table 3.

### 3.3. Body mass

There was no significant group ×time interaction, main time effect, or main group effect for body mass (RETO: Week 0 = 85.37 ± 13.43, Week 6 = 85.54 ± 13.72, Week 12 = 86.26 ± 13.76; PRP: Week 0 = 82.89 ± 12.84, Week 6 = 83.18 ± 12.90, Week 12 = 83.23 ± 13.10; PRP: Week 0 = 88.76 ± 13.71, Week 6 = 89.19 ± 13.47, Week 12 = 88.76 ± 13.57).

### 3.4. Muscle thickness

Total quadriceps muscle thickness demonstrated a significant main effect of time (F(2, 52.1) = 16.53, *p* < 0.001), with pairwise comparisons revealing increases from Week 0 to Week 6 (EMM = 0.42 cm, 95% CI [0.12, 0.54], *p* < 0.001) and Week 0 to Week 12 (EMM = 0.42 cm, 95% CI [0.26, 0.67], *p* < 0.001), but no significant change between Weeks 6 and 12. No group ×time interaction or main group effect was observed. Similarly, vastus lateralis thickness exhibited a main time effect (F(2, 52.1) = 9.12, *p* < 0.001), increasing from Week 0 to Week 6 (EMM = 0.11 cm, 95% CI [0.02, 0.21], *p* = 0.017) and Week 0 to Week 12 (EMM = 0.16 cm, 95% CI [0.06, 0.25], *p* < 0.001), with no group-related differences. In the rectus femoris, a main time effect (F(2, 52.1) = 5.72, *p* = 0.006) indicated increases from Week 0 to Week 12 (EMM = 0.13 cm, 95% CI [0.03, 0.23], *p* = 0.006), but no other significant changes. The vastus intermedius showed a main time effect (F(2, 52.1) = 6.55, *p* = 0.003), driven by Week 0 to Week 12 increases (EMM = 0.18 cm, 95% CI [0.05, 0.31], *p* = 0.003), with no other changes. Vastus intermedius data failed normality and were log transformed. Overall, there were no group differences over time as evident by no significant interactions. Mean values and SDs are presented in Table 4.

**Table 4.** Muscle thickness.

	RETO (*n* = 10)			PRP (*n* = 9)			PSP (*n* = 11)		
Muscle thickness (cm)	Week 0	Week 6	Week 12	Week 0	Week 6	Week 12	Week 0	Week 6	Week 12
Total	6.38 ± 0.95	6.43 ± 0.91	6.79 ± 0.85	6.14 ± 0.91	6.67 ± 0.88	6.70 ± 1.16	6.33 ± 1.12	6.78 ± 0.85	6.76 ± 0.89
Vastus lateralis	2.06 ± 0.40	2.07 ± 0.32	2.14 ± 0.39	1.90 ± 0.29	2.08 ± 0.37	2.17 ± 0.42	2.13 ± 0.38	2.30 ± 0.31	2.25 ± 0.40
Rectus femoris	2.30 ± 0.33	2.30 ± 0.21	2.41 ± 0.30	2.23 ± 0.33	2.32 ± 0.22	2.31 ± 0.22	2.35 ± 0.42	2.54 ± 0.33	2.54 ± 0.33
Vastus intermedius	2.02 ± 0.44	2.06 ± 0.51	2.24 ± 0.41	2.02 ± 0.45	2.28 ± 0.50	2.22 ± 0.75	1.85 ± 0.64	1.95 ± 0.55	1.97 ± 0.48

RETO = Resistance exercise training only and no additional protein; PRP = post-resistance exercise training protein; PSP = pre-sleep protein; cm = centimeters. Data presented as mean ± standard deviation.

### 3.5. Maximum strength

**Figure 2.**
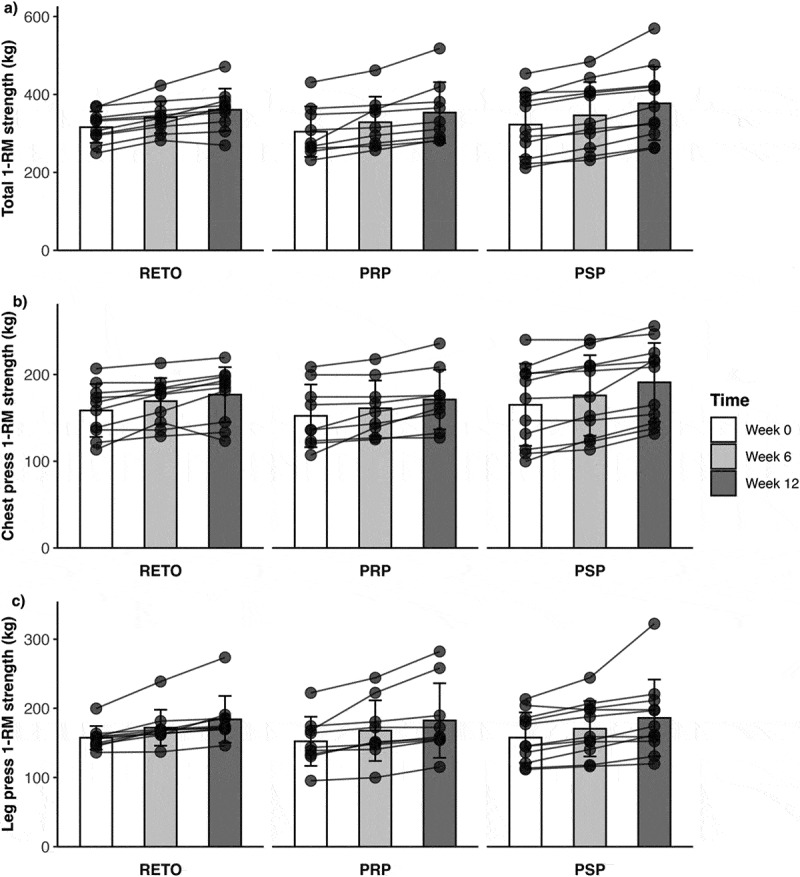
1-RM strength changes. Data presented as mean ± SD and individual values. 1-RM = one-repetition maximum, kg = kilograms. PRP = post-resistance exercise training protein (*n* = 9). PSP = pre-sleep protein (*n* = 11). RETO = resistance exercise training only and no additional protein (*n* = 10). Panel a: total (leg press plus chest press) 1RM strength. Panel b: chest press 1RM strength. Panel b: leg 1RM strength.

Total 1-RM strength exhibited a significant main effect of time (F [2 54] = 61.04, *p* < 0.001), with pairwise comparisons indicating increases from Week 0 to Week 6 (EMM = 24.3 kg, 95% CI [13.3, 35.3], *p* < 0.001), Week 6 to Week 12 (EMM = 25.0 kg, 95% CI [14.0, 36.0], *p* < 0.001), and Week 0 to Week 12 (EMM = 49.3 kg, 95% CI [38.3, 60.4], *p* < 0.001). No group ×time interaction or main group effect was observed. Similarly, chest press 1-RM demonstrated a main time effect (F [2 54] = 45.69, *p* < 0.001), increasing from Week 0 to Week 6 (EMM = 10.1 kg, 95% CI [4.68, 15.50], *p* < 0.001), Week 6 to Week 12 (EMM = 10.9 kg, 95% CI [5.50, 16.3], *p* < 0.001), and Week 0 to Week 12 (EMM = 21.0 kg, 95% CI [15.59, 26.40], *p* < 0.001), with no group-related differences. For leg press 1-RM, log-transformed data (to address non-normality) showed a main time effect (F [2 54] = 55.31, *p* < 0.001), with significant gains from Week 0 to Week 6 (EMM = 14.2 kg, 95% CI [5.52, 23.0], *p* < 0.001), Week 6 to Week 12 (EMM = 14.1 kg, 95% CI [5.39, 22.80], *p* < 0.001), and Week 0 to Week 12 (EMM = 28.3 kg, 95% CI [19.63, 37.1], *p* < 0.001). Overall, there were no group differences over time as evident by no significant interactions. Individual and group data are presented in Figure 2.

## 4. Discussion

The primary findings of this study demonstrate that consuming 40 g of supplemental protein at either PRP or PSP did not enhance RET-induced increases in quadriceps muscle thickness or maximal strength compared to RETO in untrained older adults. Contrary to our hypothesis, neither protein timing strategy elicited superior adaptations relative to RETO, despite significant increases in total daily protein intake in both supplemented groups. These results suggest that when baseline protein intake is sufficient (≥1.0 g/kg/day), additional protein supplementation – regardless of timing – may not further augment muscular adaptations in this population. Furthermore, the absence of group differences underscores that RET alone provides robust improvements in strength and hypertrophy among previously sedentary older adults, potentially overshadowing any benefits from additional, or timed, protein intake.

### 4.1. Muscle thickness

The lack of difference in quadriceps muscle thickness between all groups suggests that, under conditions of adequate baseline protein intake, neither distribution nor additional protein meaningfully influence muscle size adaptations in untrained older adults. While all groups exhibited increases in total quadriceps thickness over 12 weeks (Δ + 0.42 cm, 95% CI [0.26, 0.67]), they were similar across conditions, aligning with meta-analyses reporting inconsistent benefits of protein supplementation on muscle size in older populations [1,17]. Notably, discrepancies between measurement methods – such as muscle thickness, cross-sectional area, lean mass, fat-free mass (lean mass and bone mineral content) – may partly explain the conflicting findings in the literature, as there is poor agreement between measurements [18]. Furthermore, ultrasound-derived muscle thickness measurements are a local index of muscle growth and do not capture the heterogeneous hypertrophy patterns across muscle regions [19]. However, we measured muscle thickness for four different quadriceps muscles, all of which showed similar increases between groups, increasing the confidence that there was no difference between groups for increases in quadriceps muscle size.

Prior studies reporting null effects of pre-sleep and post-exercise protein supplementation during RET on muscle size in older adults have provided suboptimal protein doses (21 g/day) insufficient to overcome age-related anabolic resistance [11]. By contrast, our intervention delivered 40 g/serving – a dose previously shown to maximally stimulate muscle protein synthesis in older adults [12]—yet still failed to augment hypertrophy. Interestingly, the previous investigation also reported that baseline protein consumption was ≥1.0 g/kg/day. Taken together, this implies that when habitual protein intake meets or exceeds recommended thresholds (1.0–1.3 g/kg/day; [20]), further supplementation is not a limiting factor for muscle size adaptations to RET, regardless of timing, in older untrained adults. Future studies should incorporate multiple measurement modalities to resolve discrepancies in muscle growth assessments and better characterize hypertrophy responses.

### 4.2. Maximum strength

The similar improvements in maximal strength across all groups suggest that protein timing does not modulate the relationship between muscle growth and strength adaptations during initial resistance training phases in older adults. Leg press (Δ + 28.3 kg, [19.63, 37.1]) and chest press (Δ + 21.0 kg, [15.59, 26.40]) 1-RM strength improvements were consistent with prior meta-analyses of RET in older adults [14]. These early RET strength increases are likely driven by neuromuscular adaptations rather than hypertrophy, which may explain why additional protein had no additive effect. While acute evening protein intake has shown ergogenic potential for recovery and performance in younger adults [7], such benefits did not translate to long-term strength gains in our study. Furthermore, our findings align with Holwerda et al. (2018), who similarly reported no strength benefits from pre-sleep or post-exercise protein supplementation in older adults, even when providing 21 g of protein. Our study extends these observations by demonstrating that doubling the protein dose (40 g) still fails to augment strength gains. Additionally, the lack of group differences agrees with meta-analyses reporting minimal additive strength benefits from protein supplementation in older adults, even with 18–22 weeks of RET [17,21]. These findings may not necessary be age-dependent, as even in younger adults, the benefit of an additional 20 g of protein supplementation during RET was marginal (~2.5 kg over 13 weeks; [22]), suggesting that timing strategies may offer limited value across populations when total protein intake is sufficient. Collectively, these results emphasize that untrained older adults can achieve robust strength gains through RET alone, provided habitual protein intake is adequate. Prioritizing RET adherence and daily protein sufficiency – rather than precise timing – appears most critical for augmenting strength in older adults.

### 4.3. Limitations

There are several limitations to consider when interpreting the results of the current study. First, participants had a baseline protein intake above 1.0 g/kg/day, potentially diminishing the effects of additional protein supplementation. Second, muscle thickness measurements, though reliable, were limited to a single site per muscle, which may not have fully captured hypertrophy at other locations along the muscle [19]. Finally, the 12-week intervention and twice-weekly training frequency – though effective for improving hypertrophy and strength in untrained older adults – may have limited the potential to detect the effects of additional protein intake, despite evidence that longer or more frequent protocols also do not demonstrate a benefit [17,21].

## 5. Conclusion

This study demonstrates that consuming 40 g of supplemental protein either post-exercise or pre-sleep does not enhance resistance exercise training (RET)-induced improvements in muscle thickness or maximal strength compared to RET alone in untrained older adults with adequate baseline protein intake (≥1.0 g/kg/day). The absence of group differences indicates that protein timing should not be emphasized when optimizing RET in this population. Prioritizing consistent RET adherence and ensuring adequate daily protein intake (≥1.0 g/kg/day) appears more critical than acute timing strategies for untrained older adults.

## Data Availability

The datasets generated during and/or analyzed during the current study are available at https://osf.io/ae5un/?view_only=4dcee5e2845a4304936dc5f05c2b158c.
